# Abnormal Functional Connectivity of Amygdala in Late-Onset Depression Was Associated with Cognitive Deficits

**DOI:** 10.1371/journal.pone.0075058

**Published:** 2013-09-10

**Authors:** Yingying Yue, Yonggui Yuan, Zhenghua Hou, Wenhao Jiang, Feng Bai, Zhijun Zhang

**Affiliations:** 1 The Department of Neuropsychiatry, Affiliated ZhongDa Hospital and Institute of Neuropsychiatry of Southeast University, Nanjing, China; 2 Department of Psychiatry, The 4th People’s Hospital of Wuhu City, Wuhu, China; Beijing Normal University, China

## Abstract

**Background:**

Major depressive disorder (MDD) is associated with decreased function of cortico-limbic circuits, which play important roles in the pathogenesis of MDD. Abnormal functional connectivity (FC) with the amygdala, which is involved in cortico-limbic circuits, has also been observed in MDD. However, little is known about connectivity alterations in late-onset depression (LOD) or whether disrupted connectivity is correlated with cognitive impairment in LOD.

**Methods and Results:**

A total of twenty-two LOD patients and twenty-two matched healthy controls (HC) underwent neuropsychological tests and resting state functional magnetic resonance imaging (rs-fMRI). Regional homogeneity (ReHo) and FC with bilateral amygdala seeds were used to analyze blood oxygen level-dependent fMRI data between two groups. Compared with HC, LOD patients showed decreased ReHo in the right middle frontal gyrus and left superior frontal gyrus. In the LOD group, the left amygdala had decreased FC with the right middle frontal gyrus and the left superior frontal gyrus in the amygdala positive network, and it had increased FC with the right post-central gyrus in the amygdala negative network. However, significantly reduced FC with the right amygdala was observed in the right middle occipital gyrus in the amygdala negative network. Further correlative analyses revealed that decreased FC between the amygdala and the right middle occipital gyrus was negatively correlated with the verbal fluency test (VFT, *r* = −0.485, *P* = 0.022) and the digit span test (DST, *r* = −0.561, *P* = 0.007).

**Conclusions:**

Our findings of reduced activity of the prefrontal gyrus and abnormal FC with the bilateral amygdala may be key markers of cognitive dysfunction in LOD patients.

## Introduction

Late**-**onset depression (LOD), which refers to depressive syndromes as defined in the American Psychiatric Association’s Diagnostic and Statistical Manual (DSM-IV), is an important public health problem due to its high prevalence of 6.5% to 9% [1]–[3]. Different from other forms of depression, LOD is characterized by increased anhedonia, apathy, a lower rate of family history of mood disorders, non-response to initial antidepressants and a greater number of vascular risk factors. LOD is often associated with cognitive impairment, so it is often referred to as either a pseudodementia syndrome or a prodrome of dementia [4]–[8]. Episodic memory and executive function are much worse in LOD patients than in healthy controls [9]. This cognitive impairment persists even after the remission of mood symptoms [10]. However, the pathogenesis of cognitive impairment in LOD is unclear.

With the development of modern imaging techniques, more methods have been used to explore the pathogenesis of LOD. Resting state fMRI (rs-fMRI), which is noninvasive and only requires the participants to remain relaxed and still with their eyes closed during scanning, could provide new insights into how structurally segregated and functionally specialized brain networks in the low-frequency range (<0.1 Hz) of blood oxygenation level-dependent (BOLD) fluctuations are interconnected. In rs-fMRI, regional homogeneity (ReHo), which reflects the temporal changes in neural activity in brain regions, and functional connectivity (FC) analysis, which measures the correlation coefficients of all brain areas with a predefined region, have often been used. ReHo was first used to analyze imaging data by Zang [11]. In this method, Kendall's coefficient concordance (KCC) was used to measure the similarity of a time series of a given voxel to its nearest voxels in a voxel-wise manner based on the postulation that a voxel would be temporally similar to its neighbors [12], [13]. Abnormal ReHo is relevant to the changes of neural activity in distinct brain regions, and it has been used to explore changes in pathology-related brain regions. FC measures temporal correlations of spontaneous BOLD signals in different brain regions. fMRI studies have reported abnormalities in important brain regions such as the prefrontal regions, amygdala, hippocampus, thalamus, medial temporal lobes, posterior cortex and anterior cingulate cortex in MDD [14]– [17].

Alterations in resting state connectivity have been observed in MDD across multiple networks, including parts of the cognitive control network (anterior cingulate cortex, prefrontal cortex) that are involved in decision making, attention and resolving conflicts; the default mode network (posterior cingulate, precuneus, inferior parietal, medial prefrontal cortex); and parts of the affective network (orbitofrontal cortex, striatum, amygdala) that are involved in processing emotional and rewarding information [18]– [27]. Moreover, resting state network alterations in the default mode network, affective network or visual cortical areas may be virtual biomarkers for distinguishing MDD from other mental disorders [28]. In MDD, previous studies have showed decreased activation of cortical regions, including the dorsolateral prefrontal cortex, but increased activation of limbic regions such as the amygdala and medial thalamus [29]–[32]. This pattern of regional activity in MDD has led to the postulation of a putative prefrontal-amygdalar-pallidostriatal-medio thalamic mood-regulating circuit [33], [34]. All of the above data showed that abnormalities in the prefrontal cortex and amygdala will inevitably reduce emotional processing and response capabilities. Emerging evidence has suggested that patients with LOD may have structural as well as functional abnormalities in these brain areas. Structural MRI studies have consistently reported that MDD patients have larger amygdala volumes compared with non-depressed subjects and that amygdala size can be a predictor of the acute state of depression [35], [36]. Decreased volume of the right amygdala, bilateral hippocampus, right frontal lobe, orbitofrontal lobe and left temporal lobe have been reported in voxel-based morphometry analyses of LOD patients, but not in early-onset depression and healthy elderly controls [37]– [39]. In addition, studies using FC to measure signal synchronization between remote brain areas found significantly lower connectivity in MDD in paths leading from the amygdala to the orbitofrontal cortex and anterior cingulate cortex [40]. Most MRI studies, however, have focused on MDD, thus, little is known about resting state disturbances in cortico-limbic circuits in LOD patients. Here, we report abnormal activity of brain regions measured by ReHo and abnormal activity of cortico-limbic circuits detected by FC.

The first aim of this study was to investigate the function of the prefrontal cortex, amygdala and FC patterns of amygdala networks in patients with LOD using resting state fMRI. Second, we detected the relationships between abnormal brain regions and cognitive function in LOD patients.

## Materials and Methods

### Participants

This study was approved by the Medical Ethics Committee for Clinical Research of Zhongda Hospital Affiliated to Southeast University. All patients and healthy controls were gave written informed consent to participate in the study. A total of 22 LOD inpatients and 22 age- and sex-matched healthy controls were recruited. To be enrolled in our study, participants needed to meet the following inclusion criteria: (1) Participants fulfilled the diagnostic criteria for MDD using a Structured Clinical Interview by two trained and senior psychiatrists (Z. Hou and Y. Yuan) according to the Diagnostic Statistical Manual of Mental Disorder, Fourth Edition (DSM-IV); (2) Participants were first onset and medication-naïve, and the age of onset was 55 years or older; (3) Each participant’s Hamilton Depression Rating Scale (HAMD-17) score was greater than 17; (4) Participants were right-handed; (5) Participants were free of other major psychiatric disorders, including substance abuse (caffeine, nicotine and alcohol), neurodegenerative illness, severe physical illnesses and other medical illnesses causing impaired cognitive function; (6) Participants had no contraindications to MRI scanning; and (7) Participants had no cardiac or pulmonary disease that could influence the BOLD response. The inclusion criteria for HC participants are similar to LOD patients except fulfilling the diagnostic criteria for MDD and Hamilton Depression Rating Scale (HAMD-17) score was greater than 17. Diagnostic evaluations were carefully conducted on all participants, which included a clinical interview, a focused neurological and mental status exam and a demographic inventory.

### Neuropsychological measurements

All subjects underwent diagnostic evaluations, including the Hamilton Depression Rating Scale (HAMD); the Hamilton Anxiety Rating Scale (HAMA); and cognitive function testing with a neuropsychological battery that consisted of the Mini Mental State Examination (MMSE), the Auditory Verbal Learning Test (AVLT)-delayed recall, the Digit Span Test (DST-forward and backward), the Symbol Digit Modalities Test (SDMT), the Verbal Fluency Test (VFT-animal and verb) and the Trail Making Test (TMT-A and B). The detailed information about the neuropsychological assessments included the normal ranges of the scores and the reference is available in File S1. We merged the measurement of similar cognitive domain and the process is as bellows: First, all scale scores was transformed to standard Z value in order to avoid the influence of the different measurement units. Second, the scale scores of representing same domain were added up. This set of neuropsychological tests was grouped into the following domains: overall cognitive function (MMSE), memory function (AVLT-delayed recall), language (VFT), executive function (TMT-B), processing speed (SDMT, TMT-A), attention function (DST).

### Image acquisition and processing

The subjects were scanned using a General Electric 3.0 Tesla Siemens Magnetom Symphony scanner and a standard head coil. Subjects lay supine with the head snugly fixed by a belt and foam pads to minimize head motion. A Gradient-recalled echo-planar imaging (GRE-EPI) pulse sequence was set up to acquire resting state images. Scan parameters were as follows: 30 axial slices, repetition time  =  3000 ms; echo time  =  30 ms; flip angle  =  90; acquisition matrix  =  64×64; field of view  =  240 mm×240 mm; thickness  =  4.0 mm; gap  =  0 mm and 3.75 mm×3.75 mm in-plane resolution parallel to the anterior commissure–posterior commissure line. This acquisition sequence generated 142 volumes in 7 min and 6 s. All subjects were instructed to close their eyes and not to think of specific things during scanning.

### Functional image preprocessing

The preprocessing of rs-fMRI images was performed using SPM5 (http://www.fil.ion.ucl.ac.uk/spm) and REST (http://www.resting-fmri. sourceforge.net). The first ten volumes of the scanning session were discarded to allow for T1 equilibration effects. The remaining images were corrected for timing differences between slices and for motion effects (six-parameter rigid body) using a reference volume in the center of the run. Recently, many studies have demonstrated that head motion might have a systematic influence, decreasing long-distance correlations while increasing short-distance correlations on multiple types of FC analysis in rs-fMRI [41]–[44]. Therefore, participants with head motion of more than 2.5 mm of maximum displacement in any direction (x, y or z) or 2.5 degrees of angular motion were excluded from the present study. We also counted the maximum and minimum of each subject’s head curve, and the Mann Whitney U-test was performed. No significant differences were found between the two groups (*P*>0.05). In addition, the mean motion which represents the mean absolute displacement of each brain volume as compared to the previous volume and was estimated from the translation parameters in the x (left/right), y (anterior/posterior), and z (superior/inferior) directions was showed no significance difference between two groups (*P* = 0.622). The resulting images were spatially normalized into a standard stereotaxic space using a 12-parameter affine approach and an EPI template image that was resampled to 3×3×3 mm^3^ voxels and smoothed with a Gaussian kernel of 8×8×8 mm (full-width half-maximum FWHM). Ultimately, the resulting fMRI data were filtered (0.01<f<0.08 Hz) to reduce low-frequency drift and high-frequency physiological respiratory and cardiac noise. Any linear trend was then removed.

### ReHo data analysis

Regional homogeneity analysis was performed with the in-house software REST (http://www.resting-fmri.sourceforge.net). The smooth function noted above was performed after ReHo. Individual ReHo maps were generated by calculating the Kendall’s coefficient concordance of the time series of a given voxel with those of its nearest neighbors (26 voxels) in a voxel-wise manner. The Kendall’s coefficient of concordance was computed by a formula given in a previous study [11]. To reduce the effect of individual variations on the Kendall’s coefficient of concordance value, ReHo maps were normalized by dividing the averaged Kendall’s coefficient of concordance among each voxel of the whole brain. To explore the ReHo difference between LOD patients and healthy controls, a two-sample t-test was performed on the group ReHo maps in a voxel-by-voxel manner. A threshold of *P<*0.05, corrected by a Monte Carlo simulation for multiple comparisons (See program AlphaSim by D. Ward, and http://afni.nimh.nih.gov/pub/dist/doc/manual/AlphaSim.pdf) which is an algorithm that computes the probability of a random field of noise producing a cluster of a given size after the noise is thresholded at a given level and it has been widely used in fMRI field to estimate the probability of a false detection. Then an extent threshold greater than 148 cluster sizes, was applied to the resulting statistical map [45].

### Functional connectivity analyses

The functional connectivity analyses were performed with the rs-fMRI Data Analysis Tool Kit (REST, http://resting-fmri. sourceforge.net). The left and right amygdala masks were manually traced on T1-weighted 3D SPGR images, according to the literature [46], [47]. The boundaries were as follows: posterior, the anterior alveus of the hippocampus and the temporal horn of the lateral ventricle; anterior, 2 mm from the temporal horn of the lateral ventricle; superior, ventral horn of the subarachnoid space; inferior, most dorsal finger of the white-matter tract under the horn of the subarachnoid space; lateral, 2 mm from the surrounding white matter; and medial, 2 mm from the subarachnoid space. Operationally, a mouse-controlled cursor traced relevant coronal, sagittal, and axial slices. To confirm the accuracy of tracings, the amygdala was traced and calculated twice; the result showed a good correlation (left: *r* = 0.808, *P<*0.001; right: *r* = 0.79, *P<*0.001). The mean image of twice manually traced amygdala was used as the final ROI, and functional connectivity was plotted on each individual map. For every subject, a mean time series for each amygdala was separately computed as a reference time course. Cross-correlative analysis was then carried out between the mean signal change in each region and the time series of each voxel of the whole brain. A Fisher’s z-transform was applied to improve the normality of the correlation coefficients [48]. Six head motion parameters and the mean time series of global, white matter and cerebrospinal fluid signals were introduced as covariates into a random effects model to remove possible effects of head motion, global, white matter and cerebrospinal fluid signals on the results.

Within the two groups, individual z-values were entered into a one-sample t-test in a voxel-wise manner to determine the brain regions showing significant connectivity to the amygdala at *P<*0.05, corrected by Monte Carlo simulation. Two independent t-tests were performed to explore whether the functional connectivity of the amygdala was different between LOD patients and controls. The thresholds were set at a corrected *P<*0.05 (bilateral), determined by Monte Carlo simulation for multiple comparisons. It should be noted that maps from the one-sample t-test of each group were used as mask when we performed comparisons between groups, thresholded leniently at *P* = 0.05 and corrected. Masks were produced as follows: first, we performed a one-sample t-test in the LOD group and the HC group separately. Second, a Monte Carlo simulation was used to correct the significance level to 0.05. Third, only positive or negative functional connectivity was used to create the mask for the two independent t-tests separately. Therefore, two independent t-tests were performed on four different masks of bilateral amygdala (positive networks and negative networks of each amygdala).

### Statistical analysis

Two independent t-tests and Chi-squared tests were used to compare demographic performance (statistical significance was set at *P<*0.05). Analysis of covariance was used to compare cognitive function, which was closely related to cognitive level. Two independent t-tests were used to compare the imaging differences between groups by taking the above four networks as mask. Further correlative analysis between fMRI data and neuropsychological performance was then performed on the LOD and HC groups by extracting masks of the significant differences in functional connectivity between groups. Then, mean z-values of abnormal functional connectivity regions were calculated for every subject. These analyses were performed using the REST extract ROI Series (REST, by SONG Xiaowei, http://resting-fmri.sourceforge.net). Finally, Spearman's correlative analyses were performed to examine relationships between abnormal z-values and standardized neuropsychological performance scores using SPSS 18.0 software (SPSS, Inc., Chicago, IL). It deserves to be specially noted that we merged the measurements representing the same domains of cognitive field together (VFT-animal and VFT-verb, DST-forward and DST-backward, SDMT and TMT-A).

### Ethics

All patients and healthy controls were gave written informed consent to participate in the study. The design of this study was approved by the Medical Ethics Committee for Clinical Research of Zhongda Hospital Affiliated to Southeast University.

## Results

### Neuropsychological results

Compared with HC, LOD patients displayed comprehensive deficits in cognitive performance, including language, attention, executive and memory functions (see Table 1). The correlative analyses in the LOD group results showed that the HAMD total score had no correlations with cognitive functions, but the HAMA total score was negatively correlated with AVLT-delayed recall (*r* = −0.440, *P* = 0.041) and HAMD score (*r* = 0.799, *P* = 0.000). The relationship between individual cognitive measures is that MMSE is related with DST (*r* = 0.514, *P* = 0.014); AVLT-delayed recall is related with VFT (*r* = −0.428, *P* = 0.047); the VFT is related with DST (*r* = 0.454, *P* = 0.034); the DST is related with TMT-B (*r* = −0.467, *P* = 0.028) and the SDMT+TMT-A (*r* = 0.487, *P* = 0.022); the SDMT+TMT-A is related with TMT-B (*r* = −0.862, *P* = 0.000). It illustrated that the different cognitive domains are related with each other, not isolated.

**Table 1:** Demographic and neuropsychological data between LOD group and HC group.

Item	LOD(n = 22)	HC(n = 22)	Z/X^2^	P Value
Age (years)	67.55±5.47	66.59±8.61	−0.729	0.466^a^
Gender(male: female)	7:15	9:13	0.393	0.531^b^
Education level (years)	8.86±4.74	12.00±2.45	−2.251	0.024^a^
MMSE	28.59±1.44	29.32±1.09	3.890	0.028^c^
HAMA	28.41±6.84	1.55±2.18	−5.695	0.000^a^
HAMD	30.27±4.93	1.77±2.39	−5.720	0.000^a^
AVLT-delayed recall	4.09±1.27	6.64±1.71	15.552	0.000^c^
SDMT	19.73±7.80	35.91±10.27	21.431	0.000^c^
DST	10.55±1.65	13.50±1.50	22.039	0.000^c^
VFT	25.05±6.51	37.18±7.01	17.792	0.000^c^
TMT-A	106.03±27.52	81.42±19.53	19.518	0.000^c^
TMT-B	181.00±53.82	157.11±35.28	8.640	0.001^c^

LOD: late-onset depression; HC: healthy controls; MMSE: Mini mental state exam; HAMA: Hamilton Anxiety Scale; HAMD: Hamilton Depression Scale; AVLT-delayed recall: Auditory Verbal Learning Test-delayed recall; SDMT: Symbol digit modalities test; DST: Digit span test-forward and backward; VFT: Verbal fluency test-animal and verb; TMT-A: Trail making test-A; TMT- B: Trail making test- B.

a Independent-samples t-test.

b Chi square test.

c Analysis of covariance.

### ReHo results

**Figure 1:**
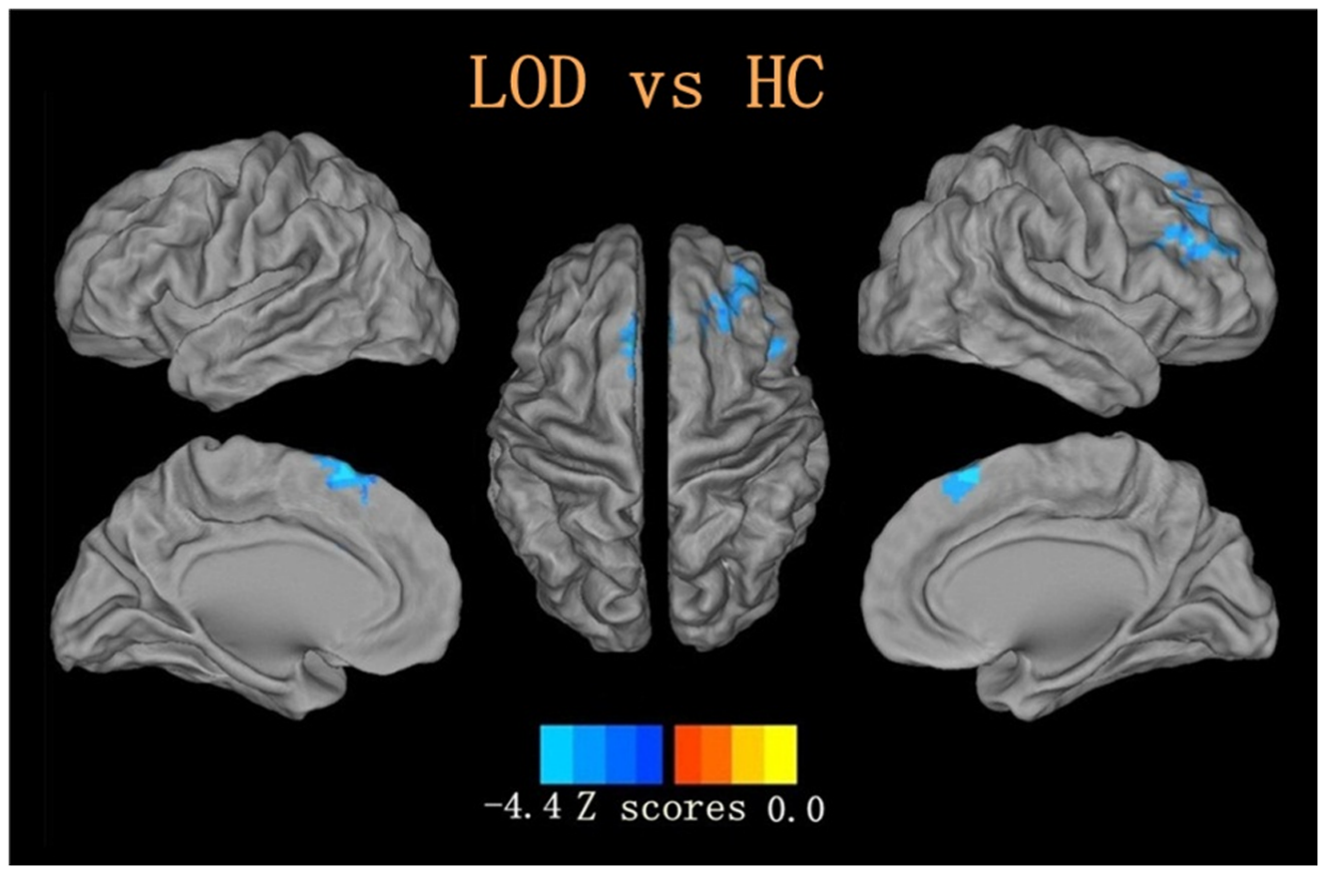
Reduced ReHo in late onset depression compared with healthy controls with voxels with *P*<0.05 and cluster size is more than 148 voxels, determined by Monte Carlo simulation,age, gender and education level as covariates.

Compared with healthy controls, a significant ReHo reduction in the right middle frontal gyrus and the left superior frontal gyrus was detected in LOD patients, taking age, gender and education level as covariates (*P<*0.05, corrected. see Table 2, Fig 1).

**Table 2:** Reduced ReHo in LOD group compared with HC group.

Brain Region	Peak MNI			Peak Z score	Cluster size
	X	Y	Z		
Middle Frontal Gyrus-R	45	36	21	−3.999	189
Superior Frontal Gyrus-L	−3	24	63	−4.4741	201

**Note:** A corrected threshold of p<0.05 determined by Monte Carlo simulation was taken as meaning there was a significantly difference between groups. R  =  right; L  =  left; cluster size is in mm^3^.

Amygdala network within-group analysis

Amygdala negative network: The one sample t-test in LOD patents showed that brain regions including the right occipital lobe and parietal lobe have negative FC with the left amygdala. Brain regions including the left parietal lobe have negative FC with the right amygdala, which is called the amygdala negative network. Similarly, in the HC group, the left amygdala negative network included the right occipital lobe, and the right amygdala negative network included the left occipital lobe and the bilateral parietal lobes.

**Figure 2:**
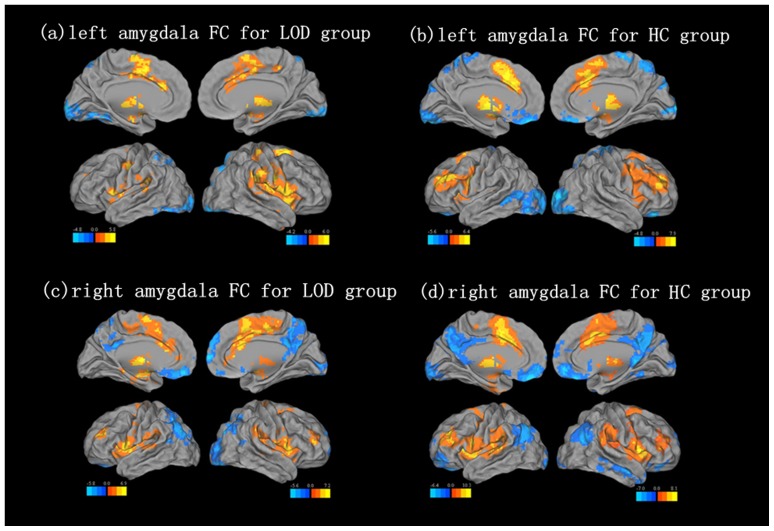
Maps of functional connectivity with Amygdala in the results of one sample t-test. Resting state left amygdala FC pattern for (a) LOD group, (b) HC group. Resting state right amygdala FC pattern for (c) LOD group, (d) HC group. Corrected with Monte Carlo simulation, P<0.05, Cluster size is more than 119 voxels.

Amygdala positive network: The one sample t-test in the LOD group showed that brain regions including the bilateral putamen and the frontal lobe have positive FC with the left amygdala. The bilateral frontal lobes showed positive FC with the right amygdala, which is called the amygdala positive network. In the HC group, the left amygdala positive network included the left cerebellum and left putamen, and the right amygdala positive network included the left cerebellum and the right inferior frontal gyrus. The results of a one-sample t-test are shown in Fig 2, and the coordinates for the centroid of each region are shown in Table 3.

**Table 3:** The result of one sample t-test in amygdala functional connectivity for LOD group and HC group.

LOD						HC					
Brain Region	Peak MNI			Peak	Cluster	Brain Region	Peak MNI			Peak	Cluster
	X	Y	Z	Z score	size		X	Y	Z	Z score	size
Negative functional connectivity with Amygdala (left)											
Occipital Lobe-R	−57	−57	−27	−4.7889	2024	Occipital Lobe-R	21	−90	6	−5.834	3041
Parietal Lobe	12	−81	51	−4.481	619						
Positive functional connectivity with Amygdala (left)											
Putamen-R	30	−12	2	7.6549	3401	Cerebellum-L	−15	−54	−24	4.3404	420
Putamen-L	−21	15	0	7.2713	2231	Putamen-L	−24	9	9	10.3615	8216
Frontal Lobe	21	3	63	5.349	1483						
Negative functional connectivity with Amygdala (right)											
Parietal Lobe-L	−33	−87	30	−5.614	3120	Occipital Lobe-L	−3	−96	−12	−5.2481	914
						Parietal Lobe-L	−33	−78	45	−6.1202	2980
						Parietal Lobe-R	45	−66	24	−4.9298	544
Positive functional connectivity with Amygdala (right)											
Frontal Lobe-L	−30	−21	6	5.0859	6207	Cerebellum-L	−27	−48	−54	5.0859	1147
Frontal Lobe-R	27	9	−9	7.6905	2655	Inferior Frontal Gyrus-R	51	15	0	10.3261	10999

**Note:** A corrected threshold of p<0.05 determined by Monte Carlo simulation was taken as meaning there was a significantly difference. Cluster size is more than 389 voxels. R  =  right; L  =  left; cluster size is in mm^3^.

### Altered functional connectivity of the bilateral amygdala

Some brain regions, including the right middle frontal gyrus and the left superior frontal gyrus, showed reduced FC with the left amygdala in the positive network and right post central gyrus in the negative network compared with control subjects, taking age, gender and education level as covariates (see Table 4).

**Table 4:** Abnormal functional connectivity of bilateral amygdala in LOD group compared with HC group.

Brain Region	Peak MNI					Peak	Cluster
	X	Y	Z			Z score	size
Decreased functional connectivity with left amygdala in positive network					
Middle Frontal Gyrus-R	33	12	45			−3.27	126
Superior Frontal Gyrus- L	−21	18	51			−3.6432	208
Increased functional connectivity with left amygdala in negative network					
Post central Gyrus-R	27	−27	75			3.2318	105
Decreased functional connectivity with right amygdala in negative network					
Middle Occipital Gyrus-R	30	−78	21			−4.0182	169

**Note:** A corrected threshold of p<0.05 determined by Monte Carlo simulation was taken as meaning there was a significantly difference between groups. R  =  right; L  =  left; cluster size is in mm^3^.

**Figure 3:**
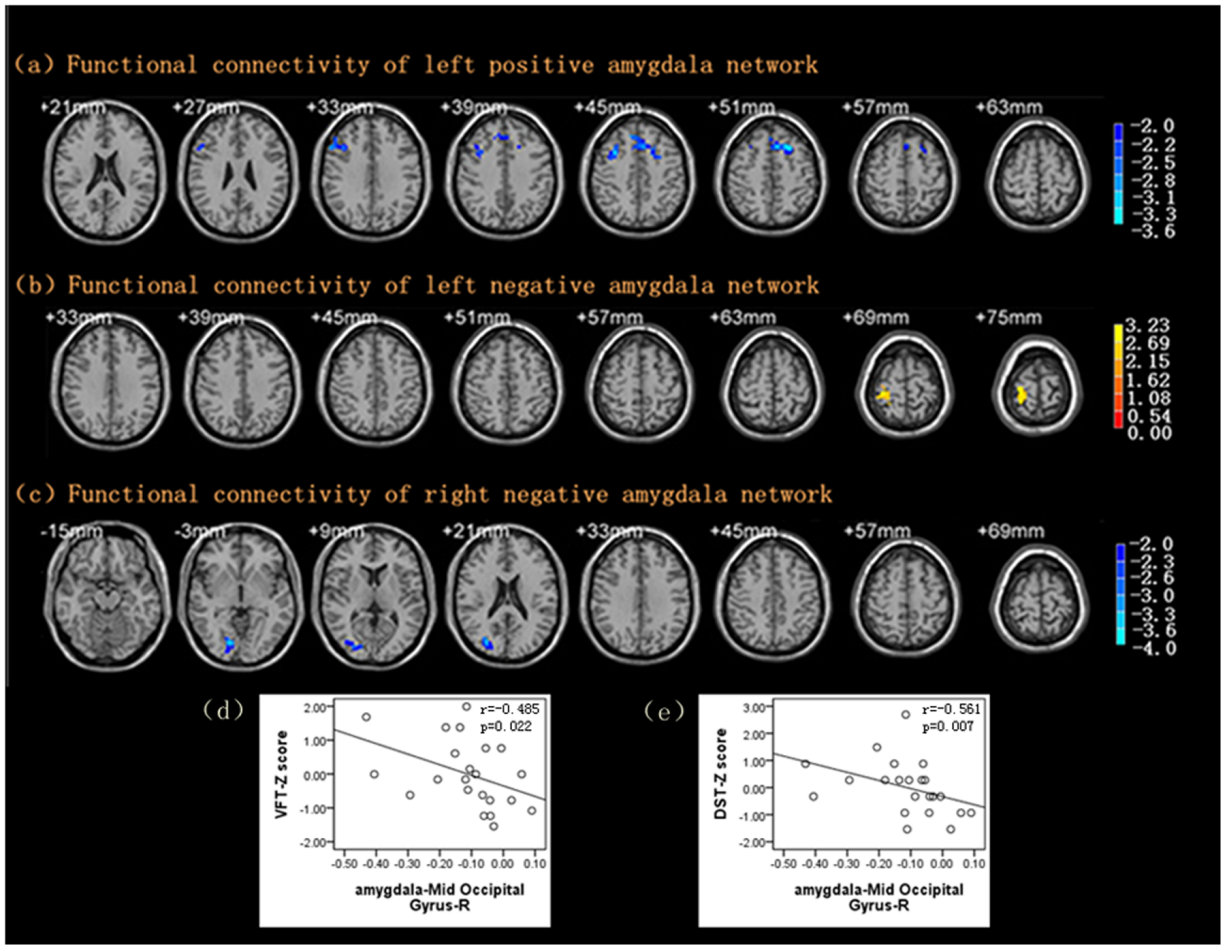
Maps of functional connectivity of Amygdala. (a) Decreased functional connectivity of left amygdala positive network in LOD compared with HC with a corrected threshold of *P*<0.05, determined by Monte Carlo simulation. Cluster size is more than 119 voxels. (b) Increased functional connectivity of left amygdala negative network in LOD compared with HC with a corrected threshold of *P*<0.05, determined by Monte Carlo simulation. Cluster size is more than 77 voxels. (c) Decreased functional connectivity of right amygdala negative network in LOD compared with HC with a corrected threshold of *P*<0.05, determined by Monte Carlo simulation. Cluster size is more than 86 voxels. (d) − (e) Significant negative correlation between FC with right amygdala and VFT/DST. VFT: Verbal fluency test; DST: digit span test; *r* = spearman’s correlation coefficient.

Similarly, FC with the right amygdala was weaker in the right middle occipital gyrus of LOD patients after controlling for age, gender and education level (see Table 4, Fig 3).

### Relationships between decreased ReHo and neuropsychological assessments

We calculated the correlation between neuropsychological assessments listed in the table 1 and abnormal ReHo as well as FC with amygdala listed in table 2/4 separately for each group. In the LOD patients, the decreased ReHo was not correlated with any neuropsychological test scores. However, the decreased ReHo in the left superior frontal gyrus was correlated with AVLT-delayed recall (*r* = 0.431, *P* = 0.045) in the HC group (see Table 5).

**Table 5:** Significant Correlations between Neuropsychological Measurements and ReHo/FC with Amygdala in HC group.

FC of Brain region	MMSE	AVLT-delayed recall	DST	VFT	SDMT+TMT-A
The correlation between decreased ReHo and neuropsychological measurements					
Superior Frontal Gyrus-L	-	0.431*	-	-	-
The correlation between FC with amygdala and neuropsychological measurements					
Middle Frontal Gyrus-R	0.464*	-	-	-	-
Post central Gyrus-R	-	-	−0.509*	-	-
Middle Occipital Gyrus-R	-	-	-	−0.467*	−0.491*

Note: this table only showed the significant correlations by Spearman's correlation. *: *P<*0.05.

### Relationships between the amygdala FC network and neuropsychological assessments

In the LOD patients, there was no correlation between any of the neuropsychological test scores and the left amygdala positive or negative networks. However, the change in FC between the right amygdala and the right middle occipital gyrus was negatively correlated with the VFT score (*r* = −0.485, *P* = 0.022) and the DST score (*r* = −0.561, *P* = 0.007) (see Fig 3). Extensive correlations between amygdala FC and neuropsychological assessments were found in the HC group. The MMSE was related to the FC with the left amygdala and the right middle frontal gyrus. The DST was related to the FC with the left amygdala and the right post-central gyrus. Furthermore, the VFT and SDMT+TMT-A scores were related to the FC with the right amygdala and the right middle occipital gyrus. However, the FC with the bilateral amygdala was not significantly correlated with the HAMD or HAMA scores in the two groups (see Table 5).

## Discussion

Using rs-fMRI to study LOD patients, three major findings were generated. First, Compared with HC, LOD patients showed decreased ReHo in the right middle frontal gyrus and the left superior frontal gyrus. Second, altered FC with the bilateral amygdala was observed in patients with LOD. Third, decreased FC between the amygdala and the right middle occipital gyrus in LOD patients was associated with cognitive impairment, but not with the severity of depression.

Although MDD is generally characterized as a mood disorder, there is increasing recognition that it is also a cognitive disorder in many older patients [49]. Comorbid depression and cognitive impairment are a particular clinical concern because they increase the rate of adverse outcomes for physical health, functional status and mortality [50]. Our results support previous studies indicating that LOD patients show multidimensional cognitive impairment related to attention, episodic memory and executive function. Moreover, longitudinal follow-up studies found that patients with remitted geriatric depression (RGD) still showed poorer cognitive function compared to healthy controls, even after the remission of mood symptoms [51].

Specific neurocognitive deficits involving attention and executive dysfunction are more common when the first episode of depression occurs in elderly patients [52]. The prefrontal cortex appears to play a role in the selective processing of affective stimuli, and impairments in its connectivity to the ventral system are associated with a more rapid reaction to sad words than to happy words in MDD patients [53]. Frontal hypometabolic activity in MDD patients can partly reverse after successful antidepressant treatment [54]. The major finding of this study is that greater resting state functional deficits were found in the prefrontal cortex of LOD patients, including the right middle frontal gyrus and the left superior frontal gyrus. The above results agree with previous studies in MDD patients. Further, correlative analysis found that abnormal activity of these brain regions was not correlated with neuropsychological deficits in LOD patients. However, we found correlations between abnormal ReHo in the prefrontal gyrus and AVLT-delayed recall scores in the HC group, indicating that changes in local brain activity are related to cognitive dysfunction.

Another interesting finding of this study is that brain regions that have decreased FC with the left amygdala include the right middle frontal gyrus and the left superior frontal gyrus in the amygdala positive network, while brain regions that have increased FC with the left amygdala include the right post central gyrus in the amygdala negative network. Previous studies suggested that the amygdala plays an important role in effective emotional regulation in humans and other animals, including implicit emotional learning and memory, emotional modulation of memory, emotional influences on attention and perception, emotion and social behavior, and emotional inhibition and regulation [55]. In MDD characterized by mood-congruent processing biases, amygdala dysfunction might be a critical neural hub that disposes to depression [56], [57]. Many studies have shown smaller amygdala volumes and elevated levels of amygdala activity [58]–[60]. These findings could be due to inadequate inhibition of the amygdala by the prefrontal centers [61]. Therefore, convergent studies provide support for abnormal FC between the prefrontal cortex and the amygdala, which are both key components of the neural systems that subserve emotional processing in MDD. A study of early-childhood-onset depression found a negative network with the amygdala that included the right post central gyrus and the right inferior parietal gyrus. Further, the study showed that lower connectivity between the bilateral amygdala and regions of the negative network correlated with weaker sadness dysregulation, which is means that reduced negative connectivity of the amygdala was associated with greater sadness dysregulation in the MDD patients with a personal history of early-childhood-onset or a maternal history of affective disorders. However, there was not a relationship between amygdala connectivity and MDD severity [62]. Our findings support the hypothesis that abnormal amygdala functional connectivity is also present in LOD patients.

However, rs-fMRI showed decreased connectivity in the middle occipital gyrus in LOD patients, which is different from previous results. In the past decades, Fujimoto *et al.* identified decreased metabolism in the frontal lobe, anterior cingulate, post-central and angular gyrus regions, whereas regions including the inferior temporal regions, occipital pole and basal ganglia showed increased metabolism using positron emission tomography (PET) in LOD patients [63]. Resting state fMRI using Cohe-ReHo, an advanced analytical method demonstrating regional spontaneous neural activity, showed significantly increased activity in the bilateral supplementary motor area and the right post-central gyrus [64]. A study comparing psychotic major depression, nonpsychotic major depression and a healthy group showed greater right occipital activation only in the nonpsychotic major depression group during a 2-back task, suggesting that increased effort is required for basic visual processing [65]. Our previous study found decreased ReHo distributed over the frontal and temporal lobes and decreased volumes of the left middle frontal gyrus and the left occipital gyrus in RGD in ApoE ε4 allele carriers [66], [67]. Furthermore, the molecular mechanism behind the neuroimaging results demonstrated a reduced number of γ-aminobutyric acid (GABA)ergic neurons in the occipital cortex, which is similar to observations in the prefrontal cortex [68]. All of the above studies showed that brain regions including the prefrontal gyrus, post-central gyrus and occipital gyrus had abnormal activity, but until now, few studies have focused on FC with the amygdala and the above mentioned brain regions. Our results revealed abnormal amygdala FC with these regions. These findings might be due to a failure to couple emotional processing and memory or due to possible inefficiency in using recollected emotional memories to regulate or cope with emotion. The results support the theory that MDD may be dependent on a distributed neuronal network consisting of cortical and limbic regions rather than on the activity of a discrete brain region.

We conducted correlation analyses between the changes of imaging indicators and neuropsychological performance in the LOD and HC groups. The results indicate an evident difference between the two groups: the HC group exhibited an extensive correlation, while little correlation was found in the LOD group. While the HC group demonstrates that there is a relationship between imaging indicators of connectivity and cognitive function, this relationship appears disrupted in LOD patients, perhaps due to neuronal degeneration reflected by brain volume losses as well as abnormal function. Our data suggest that abnormal amygdala connectivity might be involved in the psychopathology and pathophysiology of cognitive function in LOD.

The present work was an exploratory study, and technical and biological limitations inevitably exist. First, this was a cross-sectional study with a relatively small sample size, and the controls did not match the patients perfectly. To avoid the influence of age, sex and education level, we controlled these factors as covariates during the statistical analysis. Second, the resting state should not be considered a static condition because it may be associated with spontaneous and random uncontrolled cognitive processing. Although these disturbances affect FC to some extent, other studies have confirmed that rs-fMRI studies make a significant contribution to understanding brain structure and function. Third, Monte Carlo simulation was employed in the present study. The thresholds were set at a corrected P<0.05, determined by Monte Carlo simulation for multiple comparison (Parameters were: single voxel P value  =  0.05, FWHM = 8 mm, with mask. If fully corrected statistics (i.e. family-wise error or false discovery rate) was applied, no significant clusters would survive. Finally, small head movements and rotation are unavoidable even though participants were instructed not to move their heads and to rest with their eyes closed. However, we inspected each image, and patients with head movements greater than 2.5^0^ or 2.5 cm were excluded. In addition, the maximum motion and mean motion estimated from the translation parameters were showed no significance difference between two groups.

Overall, our present findings suggest that patients with LOD have low activity levels in several brain regions; moreover, abnormal resting state functional connectivity was not associated with the severity of depression, but it was related to multidimensional cognitive deficits. This study helps elucidate the pathogenesis of cognitive impairment in LOD patients, which may have important clinical implications. The changes of FC in resting state amygdala networks could be an indicator of cognitive dysfunction.

## Supporting Information

File S1 The detailed information about the neuropsychological assessments included the normal ranges of the scores and the reference is available in the online supporting information.
(DOCX)
