# Supplementary material for: Abnormal Functional Connectivity of Amygdala in Late-Onset Depression Was Associated with Cognitive Deficits
Source: PLoS One. 2013 Sep 10;8(9):e75058. doi: 10.1371/journal.pone.0075058 (PMC3769296; doi:10.1371/journal.pone.0075058)
Supplement: File S1 — The detailed information about the neuropsychological assessments included the normal ranges of the scores and the reference is available in the online supporting information. (DOCX) [file pone.0075058.s001.docx]

**Supporting information**: We provide the information about neuropsychological measurements as below:

MMSE:

Mini-Mental State Examination (MMSE) is widely used to evaluate aphasia, agnosia and apraxia- hallmarks of dementia [1]. It seems to merit consideration for wider acceptance in detecting cognitive impairment. The score of less 24/30 was recognized as cognitive impaired in the level of intermediate or above [2, 3]. The cutoff we set is as follows:

| Cutoff | Education years |
| --- | --- |
| ≥ 24 | ＞ 6 |
| ≥ 20 | ≤ 6 |
| ≥ 17 | Illiteracy |

VFT:

Verbal Fluency Test (VFT) is an index of semantic long-term memory, which is reflect the function of temporal lobe. The cut off scores of this test is 11 made by Guo [4].

AVLT:

Auditory Verbal Learning Test-delayed recall (AVLT) which is adapted from California Verbal Learning Test, is a method of memory assessment often used to distinguish mild cognitive impairment from other disease [5]. The cut off score is changed with age, 4 score in 55-59 year, 3 score in 60-69 year, 2 score in 70-79 year [6].

ADL:

Activity of Daily Sale (ADL) was first made by Lawton and Brody in the year 1969 [7]. In our study, the revision of 20 items was used and the normative data from 8 hospitals in Beijing is just as follows [8]:

|  |  | Matched by age | | Matched by education level | |
| --- | --- | --- | --- | --- | --- |
|  |  | 40-45 years | More than 75years | Illiteracy | college |
| cutoff(points) | 23 | 21 | 25 | 23 | 21 |

TMT:

Trail Making Test (TMT) which is consisted of two parts A and B was first used by Partington [9]. The cutoff of Trail making tests is shown below:

|  | 55-60 years | 61-70 years | 71-80 years |
| --- | --- | --- | --- |
| Trail making A-time | 70 | 80 | 120 |
| Trail making B-time | 190 | 220 | 280 |

DST and SDMT:

Digit span test (DST) and Symbol digit modalities test (SDMT) are used to evaluate the attention domain from China revised Wechsler Adult Intelligence Scale (WAIS-R) by Gong in 1981 [10].

1. Goldschmidt TJ, Mallin R, Still CN, et al. (1983) Recognition of cognitive impairment in primary care outpatients. southern medical journal 76: 1264-1265.

2. Folstein MF, Folstein SE, McHugh PR. (1975) “Mini-Mental State” a practical method for grading the cognitive state of patients for the clinician. J Psychiat Res 12: 189-198.

3. Zhang M. (1993) Mental assessment scale manual. Changsha. Hunan Science Press.

4. Sun Y, Guo Q, Yuan J, et al. (2007) The norm and the cut off scores of four fluency tests in the middle-aged and the old in Shanghai communities. Chinese Journal of Behavior Medicine Science 16: 714-717.

5. Delis DC. (1989) Neuropsychological assessment of learning and memory. In: Boller F, Grafman J, eds. Handbook of Neuropsychology. Vol.3. New York: Elsevier Science Publishers B. V. (Biomedical Division) 3-30.

6. Guo Q, Sun Y, Yu P, et al. (2007) Norm of Auditory Verbal Learning Test in the Normal Aged in China Community. Chinese Journal of Clinical Psychology 15: 132-134,141.

7. Lawton WP, Brody EM. (1969) Assessment of older people sele-maintaining and instrumental activities of daily living. Gerontologist 9: 179-186.

8. Han X, Feng F, Chen J, et al. (2005) Critical score on activities of daily living scale to diagnose dementia. Chinese Journal of Clinical Rehabilitation 9(37): 13-15.

9. Partington JE, Leiter RG. (1949) Partington' pathway test. The Psychological Service Center Bullentin 1: 9-20.

10. Gong Y. (1992) China revised Wechsler Adult Intelligence Scale. Hunan Map Press.
